# Colour Vision Changes across Lifespan: Insights from FM100 and CAD Tests

**DOI:** 10.3390/vision8030053

**Published:** 2024-09-06

**Authors:** Renārs Trukša, Sergejs Fomins, Zane Jansone-Langina, Laura Tenisa

**Affiliations:** 1 Department of Optometry and Vision Science, Faculty of Science and Technology, University of Latvia, LV-1004 Riga, Latvia; zane.jansone@lu.lv (Z.J.-L.); tenisalaura2000@gmail.com (L.T.); 2 Institute of Solid-State Physics, University of Latvia, LV-1063 Riga, Latvia; sergejs.fomins@cfi.lu.lv

**Keywords:** chromatic sensitivity, colour vision tests, colour vision deficiency, colour opponent channel

## Abstract

(1) Background: in this research study, colour vision was evaluated in individuals aged 19 to 70 years with and without red–green colour vision disorders. (2) Methods: study participant colour vision was assessed with anomaloscope, HRR, FM100 hue, and CAD tests. (3) Results: No significant correlation was found between participant age and chromatic sensitivity of the red–green colour opponent channel. However, a decrease in blue–yellow colour opponent channel chromatic sensitivity was confirmed with the FM100 hue test and CAD test. Analysis of FM100 hue test error scores across age groups revealed a decline in chromatic sensitivity in the short-wave region of visible light with increasing age. Comparison of the colour-deficient individual results of the CAD and anomaloscope tests confirmed that CAD test sensitivity and specificity reaches 100%. However, some individuals with deutan-type deficits were misclassified as having protan-type deficits. This study confirmed the effectiveness of the FM100 test in identifying individuals with moderate to severe colour vision deficits, with sensitivity and specificity rates of 81.25% and 95.38%. (4) Conclusions: It was found that the FM100 hue test effectively identifies individuals with moderate and severe red–green colour vision deficiencies. On the other hand, individuals with mild colour vision deficiencies may go undetected with the FM100 hue test.

## 1. Introduction

According to Birch, approximately 4% of the population is affected by red–green colour vision deficiencies [1]. Among women, red–green colour vision deficiency occurs less frequently at 0.5%, while in the male population, the incidence of red–green deficits reaches 8%. It is crucial to conduct colour vision examinations across different age groups to establish appropriate norms for each group. Research indicates that chromatic sensitivity typically improves in the age group of 18–30 years, remains relatively stable in the 30–40 age group, and decreases after 40 years [2,3,4,5]. The decline in chromatic resolution after 40 years is attributed to increased light scattering and absorption in the crystalline lens [6,7,8], reduced light absorption efficiency in cones [9,10], and reduced overall count of cone photoreceptors in the foveolar region [11], as well as a decrease in ganglion cells in the retina [12]. In addition to normal ageing processes, conditions such as diabetes mellitus [13,14], glaucoma [15], and cataracts [16] have been linked to reduced chromatic resolution compared to age-appropriate norms. Optometrists and ophthalmologists commonly use pseudo-isochromatic tests like HRR and Ishihara to evaluate colour vision, as these tests offer high sensitivity and specificity in detecting colour vision deficiencies [17]. While colour vision deficiency tests are valuable tools, they do have limitations. One such limitation is the possibility of individuals learning how to perform the tests correctly. Additionally, stimuli used in the tests to test specific types of colour vision deficiency belong to a particular type of confusion line [18]. It is vital to recognize that not all individuals with a particular type of colour vision deficiency can be accurately represented by a single confusion line. Davidoff et al., 2016, reported that certain red–green dichromats perform significantly better on the HRR test compared to other red–green dichromats [19]. Computerized colour vision tests, such as the CAD test, address the limitations of conventional colour vision tests by utilizing psychophysical methods to assess chromatic sensitivity thresholds across multiple directions in colour space. This approach contrasts with the restricted number of colour directions examined by tests like the HRR test. The CAD test allows for the evaluation of the chromatic resolution of the S cones through the measurement of chromatic sensitivity in four directions in colour space. Additionally, it assesses the chromatic sensitivity of L and M cones across six directions in colour space [20]. Research conducted using the CAD test has revealed that individuals diagnosed with conditions such as glaucoma exhibit a reduction in chromatic sensitivity within the red–green colour opponent channel [21]. Similarly, individuals with disorders like diabetes display diminished chromatic sensitivity in the blue–yellow colour opponent channel [22]. Despite the CAD test’s many benefits, it is still not commonly accessible. In addition to aiding in the diagnosis of colour vision abnormalities, the FM100 hue test can be used to assess colour vision in a range of medical conditions. The FM100 hue test allows for the monitoring of colour vision changes over time and the identification of instances where colour vision may be compromised. The FM100 hue test’s sensitivity and specificity depending on criteria reach 100% and 83% [23], or 89% and 100% [24], which highlights test suitability for practical colour vision assessments. By analysing the FM100 hue test cap sequences chosen by the patient, colour alterations indicative of specific diseases can be recognized, as well as the type of colour vision loss.

## 2. Materials and Methods

### 2.1. Participants

Through the University of Latvia’s network, potential research participants were invited to complete a questionnaire that included general inquiries as well as specific questions about eye and general health conditions, daily medication use, reliance on refractive correction, difficulties in distinguishing coloured stimuli, and any family history of colour vision deficits. Eligibility for the study was limited to participants free from eye and general diseases and those not taking medications known to affect colour perception. Ultimately, 146 individuals aged 19 to 70 participated, among whom 11 were identified as having red–green colour vision disorders.

### 2.2. HRR Test

The colour vision of all study participants was evaluated using the HRR pseudo-isochromatic plate test, which assesses chromatic sensitivity across six directions in colour space. This test specifically examines protan, deutan, and tritan deficiencies along two colour directions for each type. To prevent the inclusion of participants with acquired colour vision deficits, each eye was tested separately using the HRR test. This colour vision assessment is vital, as discrepancies between the results of the two eyes can point to acquired colour vision deficiency. Colour vision assessment with the HRR test was concluded if a participant made no errors on any screening panels; however, if errors occurred, further examination with diagnostic plates was conducted. This study only includes results from participants who exhibited an equal number of errors with both eyes. Colour vision assessments took place in a room illuminated by fluorescent lamps, with an intensity of 600 lux.

### 2.3. FM100 Hue Test

The FM100 hue test consists of four sets of coloured caps: the first set contains 22 caps, while the remaining three each have 21 caps. Each kit features two fixed anchor caps at either end, designed to remain stationary. Participants arrange the coloured caps so that adjacent ones are as similar as possible, creating a smooth gradient from one anchor cap to the other. The test is conducted in a light box equipped with a standard D6500 light source, providing an illumination intensity of 1250 lm. Participants completed the FM100 hue test under binocular viewing conditions, with no time limits. Before starting, they were instructed to indicate their readiness. Once they finished arranging the caps, they confirmed that the sorting was completed. The stopwatch was activated when participants signalled their readiness to start the test and stopped upon their announcement of completion. The average completion times for the first, second, third, and fourth sets were 2 min and 14 s, 2 min and 46 s, 2 min and 17 s, 2 min and 10 s respectively. Overall, participants required an average of 9 min and 26 s to finish all four sets. Individual FM100 hue test set completion times varied significantly, ranging from 46 to 552 s, while total execution times spanned from 229 to 1761 s. This study revealed that participants aged 30–39 and 40–49 completed the FM100 test more swiftly than those aged 19–29 and 50–70. Statistically significant differences in completion speed were found exclusively between the 19–29 and 30–39 age groups for the fourth set of the FM100 hue test. The FM100 hue test coloured cap arrangements were analysed by the method proposed by Smith et al., which, based on the FM100 hue test colour cap arrangements, enables the evaluation of the resolution of red–green and blue–yellow colour opponent channels [25]. We also calculated partial error scores for each of the 10 colour categories.

### 2.4. Anomaloscope Test

The anomaloscope test is widely recognized as one of the most accurate methods for identifying and differentiating red–green colour vision deficits. The anomaloscope test can detect the absence of L and M cone function or changes in L and M functions. Each participant underwent the anomaloscope test monocularly, with each eye tested separately. Room illumination was maintained below 10 lux. Additional illumination was provided by a computer monitor used alongside the Oculus HMC anomaloscope. Participants matched the stimuli in both semi-fields of the anomaloscope by adjusting the luminance of the yellow light source and the ratio of the red and green light sources. After completing the manual matching task for each eye, the width and midpoint of the matching range were measured three times for right and left eyes. Before each anomaloscope matching range and midpoint measurement, the yellow light source intensity was manually adjusted based on each participant’s anomaloscope manual matching procedure results.

### 2.5. CAD Test

**Figure 1:**
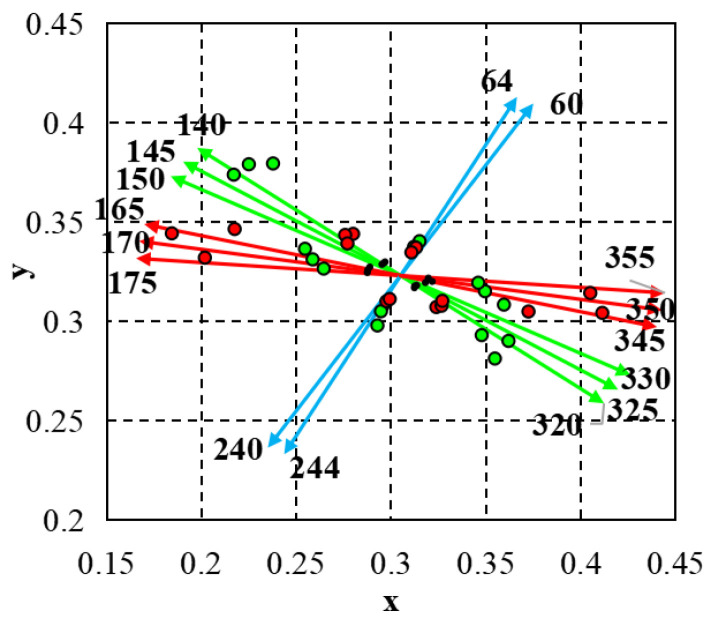
CAD test colour directions. Average chromatic thresholds of normal trichromats (see black dots), moderate protan (see red dots), and deutan (see green dots) chromatic thresholds. Red, green, and blue arrows represent the directions in colour space where chromatic stimuli are primarily detected by L, M, and S cones, respectively.

The CAD test is widely used in colour vision screening and research due to its ability to accurately assess chromatic resolution thresholds across various directions in colour space (Figure 1). This quality of CAD tests enables the monitoring of changes in colour vision over a person’s lifetime, as well as the identification of decreases in chromatic sensitivity due to eye and general diseases, or congenital colour vision deficiencies. The test was performed in a room with an illumination of 10 lux or less, under binocular viewing conditions at a distance of 1.40 m. Prior to the examination, the distance between the participant and the monitor displaying the CAD test stimuli was verified. To reduce potential human and machine errors, participants were instructed to indicate the direction of the stimulus motion by pressing a button on a remote only after the demonstration of the test stimuli had finished.

## 3. Results

To examine the influence of age on colour vision, participants were divided into five age categories: 19–29, 30–39, 40–49, 50–59, and 60–70 years. To compare the outcomes of the CAD and FM100 tests across age groups, the Games–Howell post hoc test was used. The Games–Howell post hoc procedure is recommended in cases when assumptions of equal variances and sample sizes are not met, as it does not necessitate these assumptions to be fulfilled. Research by Sauder and DeMars [26] has indicated that the Games–Howell post hoc test slightly outperforms other alternatives in terms of statistical reliability.

### 3.1. Anomaloscope Test

In this study, the relationship between the anomaloscope matching range and CAD RG chromatic sensitivity thresholds was investigated. The long standing hypothesis is that a narrower matching range indicates a higher colour resolution. The analysis did not reveal statistically significant differences in the matching range width between right and left eyes of individuals without colour vision deficits ($t=0.21<t_{0.05,126}=1.98$). The linear regression model did confirm a statistically significant relationship between the anomaloscope matching range width and CAD test RG thresholds ($t=2.26>t_{0.05,122}=1.98$). However, the model only accounted for 20.1% of the variation between the matching range width and CAD RG thresholds. Comparison of the right eye matching range width between age groups did not reveal statistically significant differences. Nevertheless, statistically significant differences were observed in the matching range width of the left eye between the age groups 19–29 (M = 3.79, SD = 1.38) and 60–70 (M = 6.52, SD = 2.32), *p* = 0.047. Additionally, no significant differences were found in the matching range width between right and left eyes among individuals with red–green colour vision deficits ($t=1.29<t_{0.05,16}=2.12$). In case of colour-deficient individuals, a statistically significant relationship between matching range width and CAD RG thresholds was not confirmed ($t=2.07<t_{0.05,15}=2.13$).

### 3.2. CAD Test—Normal Trichromats

**Figure 2:**
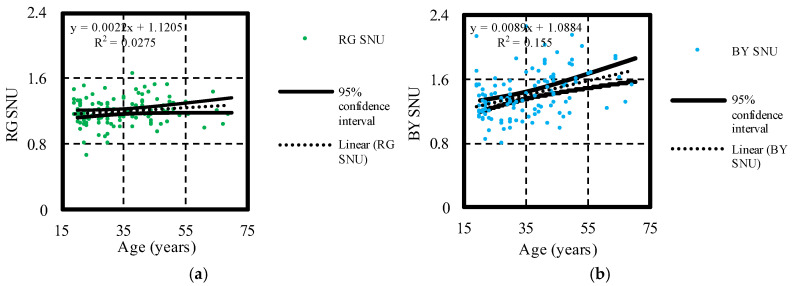
In graphs (**a**,**b**) the relationships between study participants age and chromatic sensitivity thresholds RG and BY are presented.

The findings of this study resulted in the exclusion of a few participants due to a significant decrease in chromatic sensitivity thresholds RG (*n* = 1), BY (*n* = 3), RG and BY (*n* = 1) on the CAD test according to norms suggested by the literature. Interestingly, these individuals exhibited changes solely on the CAD test and not with other colour vision tests. The participants’ performance on the CAD test during routine colour vision exams is determined by two parameters—RG and BY chromatic thresholds. Chromatic sensitivity thresholds RG and BY represent normalized chromatic sensitivity threshold values calculated in the colour directions corresponding to 67 and 334 degrees in the CIExy colour space [5]. In the colour direction BY, L and M cone excitation does not differ from L and M cone excitation by achromatic background, which enables the examination of S cone chromatic sensitivity thresholds. Conversely, in the RG colour direction, S cone excitation does not differ from S cone excitation by the achromatic background. This allows for the separate assessment of L and M cones, isolating their responses from those of the S cones. No statistically significant linear relationship was found between the age of study participants and chromatic sensitivity thresholds RG measured by the CAD test ($t=1.83<t_{0.05,119}=1.98$). On the other hand, a statistically significant relationship between age and BY chromatic thresholds ($t=4.67>t_{0.05,119}=1.98$) was found. However, the linear regression model only accounts for 15.50% of the variability in BY chromatic sensitivity thresholds (Figure 2).

BY chromatic sensitivity thresholds with CAD testing is assessed by measuring chromatic sensitivity along colour directions 60, 64, 240, and 244. Chromatic sensitivity of M cones is evaluated by measuring chromatic sensitivity along colour directions 140, 145, 150, 320, 325, and 330. Similarly, the chromatic sensitivity of L cones is assessed by measuring the chromatic sensitivity thresholds in colour directions 165, 170, 180, 345, 350, and 355. When comparing chromatic sensitivity thresholds in the colour directions where L and M cones are most sensitive across different age groups, no statistically significant differences were found. However, in directions where the sensitivity of the blue–yellow opponent channel is highest, it was found that along colour direction 60, the age group 30–39 (M = 0.0144, SD = 0.03) had lower thresholds than the age group 50–59 (M = 0.0193, SD = 0.004), *p* = 0.034; along colour direction 64, the age group 50–59 (M = 0.0188, SD = 0.0030) had higher thresholds than age groups 19–29 (M = 0.0146, SD = 0.0033), *p* = 0.011, and 30–39 (M = 0.0152, SD = 0.0035), *p* = 0.042; along colour direction 240, the age group 50–59 (M = 0.0187, SD = 0.0034) had lower chromatic sensitivity than age groups 19–29 (M = 0.0140, SD = 0.0027), *p* = 0.013, and 30–39 (M = 0.0139, SD = 0.0031), *p* = 0.011; and along colour direction 244 the age group 19–29 (M = 0.0131, SD = 0.0026) had lower thresholds than age groups 30–39 (M = 0.0150, SD = 0.0034) *p* = 0.041, 40–49 (M = 0.0153, SD = 0.0038), *p*= 0.0461, and 50–59 (M = 0.0183, SD = 0.0041), *p* = 0.021. Statistically significant differences within age groups were found only in cases when chromatic sensitivity thresholds that correspond to red–green and blue–yellow colour opponent channels were compared; in the rest of the cases there were no statistically significant differences except between colour directions 175 (M = 0.0051, SD = 0.0009) and 330 (M = 0.0043, SD = 0.0010), *p* = 0.0039, in the age group 19–29. The comparison of chromatic sensitivity thresholds between the red–green and yellow–blue opponent channels reveals that sensitivity of the red–green channel does not significantly decline with age. In contrast, age appears to play a substantial role in the decline of sensitivity in the blue–yellow channel.

### 3.3. FM100 Hue Test—Normal Trichromats

**Figure 3:**
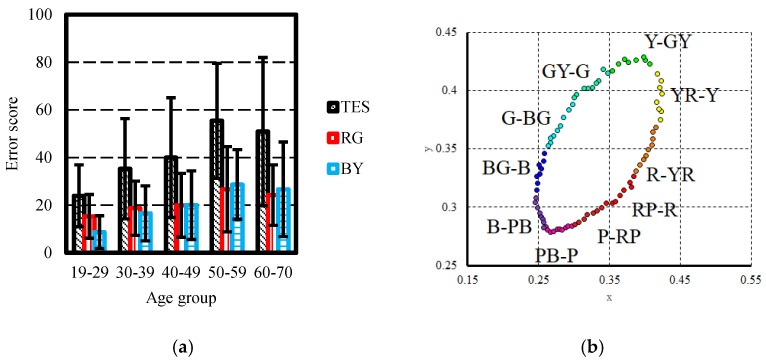
(**a**)—study participant TES, RG, and BY scores. (**b**)—FM100 hue test colour cap coordinates and colour categories.

The FM100 hue test results of the study participants were analysed using the method proposed by Kinnear [27] to calculate the total error score (TES). Additionally, partial error scores for the RG and BY axes were calculated using the method proposed by Smith et al., 1985 [25]. The RG partial error score was determined by summing the error values for colour caps numbered 13–33 and 55–75, while the BY partial error score was calculated by summing the error values corresponding to the colour caps numbered 1–12, 34–54, and 76–85. When comparing the average values of the TES scores of study participants, statistically significant differences were found between the age group 19–29 (M = 23.96, SD = 12.98) and age groups 40–49 (M = 39.97, SD = 25.12), *p* = 0.013, and 50–59 (M = 55.40, SD = 24.14), *p* = 0.017. The results of the FM100 hue test indicate that the RG partial error score values do not differ significantly between age groups. However, the BY partial error score values differ significantly between the age group 19–29 (M = 8.67, SD = 6.89) and age groups 30–39 (M = 16.57, SD = 11.56), *p* = 0.013, 40–49 (M = 20.00, SD = 14.39), *p* = 0.001, and 50–59 (M = 28.70, SD = 14.62), *p* = 0.014 (Figure 3a).

**Figure 4:**
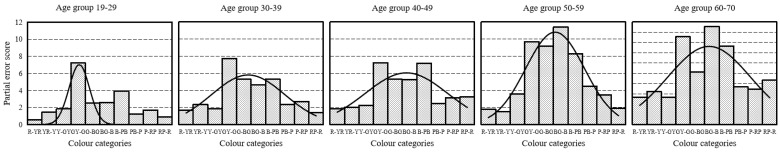
This study examined the partial error scores of research participants across 10 colour categories in the FM100 hue test. Analysis of the data revealed a notable trend wherein participants displayed an increase in errors within the short-wave spectrum of visible light as their age increased. This observation suggests a potential age-related decline in colour discrimination ability, particularly in the perception of colours within the blue and violet range.

Upon comparing the partial error scores in individual colour categories (Figure 3b), it was found that there were no statistically significant differences in partial error scores between age groups in the colour categories R-YR, YR-Y, Y-GY, GY-G, PB-P, and P-RP. However, statistically significant differences were observed in partial error scores in the colour category BG-B between the age group 19–29 (M = 2.58, SD = 3.13) and age groups 40–49 (M = 5.24, SD = 3.89), *p* = 0.015, and 50–59 (M = 11.40, SD = 7.31), *p* = 0.029; in the colour category B-PB between the age group 19–29 (M = 3.92, SD = 3.51) and age groups 40–49 (M = 7.18, SD = 4.31), *p* = 0.006, and 50–59 (M = 8.30, SD = 3.43), *p* = 0.02. Also, significant differences were found in the colour category G-BG between age groups 19–29 (M = 2.54, SD = 3.11) and 40–49 (M = 5.30, SD = 4.50), *p* = 0.028; and in the colour category RP-R between age groups 19–29 (M = 0.92, SD = 1.78) and 40–49 (M = 3.24, SD = 3.83), *p* = 0.018. To examine age effect on colour resolution average partial error scores in individual colour categories within each age group were fitted with a Gaussian function using the Matlab “Curve fitting tool”. Analysis revealed a clear pattern in the number of errors made by participants across different colour categories. It was found that as participants’ ages increased, individuals tend to make more errors when arranging colour caps that correspond to colour categories BG-B, B-PB, G-BG, and RP-R. This finding is consistent with expectations, as the absorption of light in the short wavelength range significantly increases with age (Figure 4).

### 3.4. FM100 Hue and CAD Tests—Anomalous Trichromats

**Figure 5:**
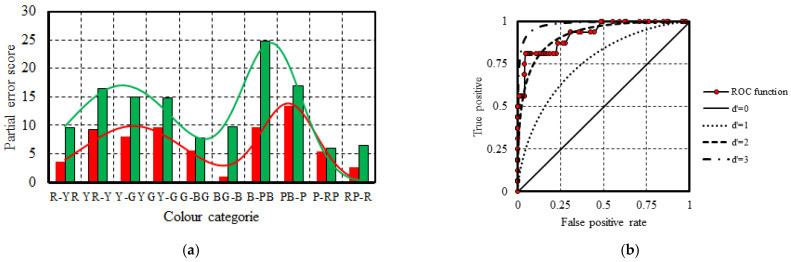
(**a**)—displays the partial error scores of individuals with red–green colour vision deficiency, protan (represented by red bars) and deutan (represented by green bars). (**b**)—illustrates the ROC function of the FM100 hue test.

Using the anomaloscope test Oculus HMC, it was found that 16 participants exhibited colour vision deficits, 5 individuals identified as protanomals, and 11 as deuteranomalous. To assess the sensitivity and specificity of the FM100 hue test, an ROC function (Figure 5b) based on study participants’ RG partial error scores (Figure 5a) was constructed. d’ was calculated by equation d’ = z(H) − z(FA), where H and FA are the hit and false alarm rates, and z is the standard normal distribution function. Analysis of the ROC function indicates that setting the RG partial error score criteria between 52 and 55 yields a d’ value of 2.58, with sensitivity and specificity at 56.25% and 100%, respectively. Alternatively, selecting the RG partial error score range of 40–41 as the criterion results in a d’ value of 2.57, achieving sensitivity and specificity levels of 81.25% and 95.38%. Nevertheless, while the former criteria have a greater d’ value, the latter criteria would be more useful in clinical practice. When comparing the performance of colour-deficient individuals in the CAD and anomaloscope tests, it was observed that the CAD test achieved 100% sensitivity and specificity. However, four deuteranomalous individuals were misclassified as having protan-type colour vision disorders, while all individuals with protan deficiencies were correctly identified.

## 4. Discussion

In the scope of this research, it was confirmed that, in general, chromatic sensitivity thresholds tend to increase with age. However, the study results suggest that age is one of several potential factors influencing colour vision. When evaluating the performance of research participants using the FM100 hue test, differences in TES scores were observed between the youngest group and the rest of the participants, except for individuals aged 60 to 70 years. Within the scope of this study, differences in performance on the FM100 hue test between younger and older groups could not be definitively confirmed, as the Games–Howell method provides a conservative estimate for relatively small sample sizes. In this research, eleven participants aged 60–70 were assessed with the CAD test, and nine participants’ colour vision was examined with the FM100 hue test. Repeating the analysis using *t*-test and assuming unequal variances, significant differences in TES scores between younger and older participant groups were found (*t* = 2.56 > *t*_0.05,9_ = 1.83). The performance of study participants in the CAD test suggests that the resolution of the red–green colour opponent channel does not significantly change with increasing age. When comparing performance across age groups in the CAD test, specifically in colour directions where L and M cones are most sensitive, no statistically significant differences were found in any of the 12 colour directions between any two age groups. The findings here contradict other studies where, due to normal aging processes, decline in red–green colour opponent channel chromatic sensitivity is confirmed. Comparing normal trichromat RG partial error scores between all age groups, statistically significant differences were not found. Significant differences were observed among age groups when analysing the chromatic sensitivity thresholds identified by the CAD test in colour directions where the blue–yellow colour opponent channel is most sensitive. Particularly, significant differences were found between the age groups 19–29 and 30–39, 19–29 and 50–59, and 30–39 and 50–59 in colour directions 60, 64, 240, and 244. Regarding the partial error score BY measured with the FM100 hue test across age groups, statistically significant variances were found between the 19–29 age group and all other age groups except for the 60–70 age group. Furthermore, upon comparing the chromatic sensitivity threshold values within age groups determined by the CAD test, it was evident that the threshold values associated with the red–green colour channel consistently exhibited lower values across all age groups compared to the chromatic sensitivity thresholds in directions related to the yellow–blue colour opponent channel. The study results indicate that, with increasing age, there is a notable decline in chromatic resolution within the short-wave spectrum of visible light. Chromatic sensitivity decline can be attributed to the increased light absorption in the short-wave spectrum by the crystalline lens. Lens light transmittance measurements in different age groups suggest that transmittance of the crystalline lens gradually decreases up to the age of 60. However, after the age of 60, there is a significant rise in light absorption in the crystalline lens [6,7,8]. A notable increase in chromatic sensitivity thresholds in individuals over 60 compared to younger individuals has been mentioned in previous studies [2,3]. Findings in this study suggest that the FM100 hue test might serve as a potential alternative for examining colour vision; however, its sensitivity falls short of that of the CAD test. This study revealed that individuals with mild red–green colour vision deficiencies might go unnoticed if the identification criteria are established at an RG partial error score of 40–41 units. A detailed analysis of the partial error scores from the FM100 hue test reveals that individuals with red–green colour vision deficiencies make significantly more errors than normal trichromats when arranging coloured caps in the B-PB and PB-P colour categories. The findings indicate that those with deutan colour vision deficiency make more errors in the B-PB category compared to individuals with protan deficiencies or normal colour vision. Deutan deficiency can be identified with 81.82% sensitivity and 94.12% specificity, if the partial error score in the B-PB colour category exceeds 16 units. Among the five individuals identified with protan deficiency, only one recorded 26 error units in the B-PB category, while the other four had 11 error units or fewer. Protan deficiency can be diagnosed by evaluating error scores in the PB-P category, where a partial error score exceeding 12 units indicates protan deficiency with 80% sensitivity and 85.21% specificity. Notably, six out of eleven individuals with deutan deficiencies in the PB-P category exhibited fewer than 12 error units. When assessing the combined sensitivity and specificity of the FM100 hue test across both the B-PB and PB-P categories, the FM100 hue test achieved a sensitivity of 96.36% and a specificity of 80.2%, resulting in a d’ value of 2.65, which surpasses the d’ value of 2.57 obtained when analysing only the RG partial error score. These results suggest that examining error values in the B-PB and PB-P colour categories can be an effective method for identifying red–green colour vision deficits. This research highlights the potential for developing a colour arrangement test that incorporates caps from both categories, which could serve as a valuable diagnostic tool for optometrists and ophthalmologists in evaluating red–green colour vision deficiencies.

It is observed that individuals with mild red–green colour vision deficiencies in most cases perform as well as colour normal individuals in colour arrangement tests, yet mild protanomalous individuals are prone to more errors than expected [28]. A few red–green colour-deficient individuals in the CAD test exhibited RG threshold values of 8.05, 6.55, and 6.11, which are 3.39, 2.76, and 2.57 times higher than the upper limit of normal trichromacy in the CAD test, and FM100 hue test RG scores of less than 41. Additionally, two participants in the CAD test displayed RG threshold values of 4.49 and 3.12, with their FM100 hue test RG partial error score reaching 45 units. The results above point to significant differences between anomalous trichromats on CAD and FM100 hue tests. Previous research has found that, in the case of anomalous trichromats, spectral sensitivities of cones and chromatic discrimination are not directly related [29]. Recent findings suggest that chromatic signals in anomalous trichromats may be amplified in the visual cortex, potentially partially explaining the significant differences in chromatic discrimination among anomalous trichromats [30].

## 5. Conclusions

The results of this study demonstrate that the FM100 hue test is effective in identifying individuals with moderate and severe red–green colour vision deficiencies. However, its performance in detecting mild deficits falls short. Statistical analysis of CAD RG chromatic sensitivity thresholds and FM100 hue test RG partial error scores do not indicate a decline in red–green colour opponent channel sensitivity with age, contrary to previous findings. The lack of correlation between age and an increase in red–green chromatic sensitivity thresholds in this study may be attributed to the exclusion of participants who reported eye or other diseases. Conversely, a noticeable rise in chromatic sensitivity threshold values for the blue–yellow colour opponent channel was observed. Insights gathered from interactions with study participants suggest that the CAD test could serve as an alternative to the anomaloscope test due to its simpler instructions.

## Data Availability

The original contributions presented in the study are included in the article material, further inquiries can be directed to the corresponding author.
